# The Impact of Fibular Fixation Method on Pilon Fracture Healing

**DOI:** 10.3390/jcm14020358

**Published:** 2025-01-09

**Authors:** Anthony Perugini, Scott Hyland, James Iandoli, Zachary Hill, John Peabody, Daniel DeGenova, Mallory Faherty, Benjamin Taylor

**Affiliations:** 1 OhioHealth, Department of Orthopedics, Columbus, OH 43228, USA; scott.hyland@ohiohealth.com (S.H.); jack.iandoli@ohiohealth.com (J.I.); tucker.peabody2@ohiohealth.com (J.P.); daniel.degenova@ohiohealth.com (D.D.); 2 OhioHealth Orthopedic Trauma and Reconstructive Surgeons, Grant Medical Center, Columbus, OH 43215, USA; zack.hill2@ohiohealth.com (Z.H.); benjamin.taylor@ohiohealth.com (B.T.); 3 OhioHealth Research Institute, Riverside Methodist Hospital, Columbus, OH 43214, USA; mallory.faherty@ohiohealth.com

**Keywords:** pilon, ankle trauma, fibular fixation, post-traumatic arthritis

## Abstract

**Background:** Pilon fractures are associated with high-energy injuries, and there is presently much debate as to optimal fixation strategies and timing of intervention. There is little evidence comparing the type of fibular fixation during pilon fracture fixation. The purpose of this study was to compare fibular fixation methods in complex pilon injuries as it relates to pilon union rates and development of post-traumatic arthritis. **Methods**: This was a retrospective review from an urban Level 1 trauma center from January 2009 to May 2019, including patients age ≥ 18 who sustained a pilon fracture with an associated fibula fracture. Patients were allocated into one of three groups based on fibular fracture treated with plating, intramedullary device, or no fixation. Radiographic analysis was performed postoperatively and at final follow up to evaluate for tibial or fibular nonunion, malunion, talocrural angle, and ankle Kellgren–Lawrence grade. **Results**: Of the 107 patients in this study, 42 underwent surgical fixation of their fibular fracture. There were no differences with respect to tibial or fibular union rates amongst the three groups. Furthermore, there were no differences in the presence of radiographic ankle arthritis at final follow up. However, Kellgren–Lawrence arthritis grading did appear to be a more severe grade in patients who did not undergo fibular fixation (*p* = 0.001). **Conclusions**: Fibular intramedullary fixation does not appear to influence tibial or fibular nonunion rates as compared to plating in complex pilon injuries.

## 1. Introduction

Pilon fractures are injuries involving the distal tibial plafond, which is the weightbearing surface of the distal end of the tibia, and classically occurs secondary to an axial compression mechanism [1]. These fractures account for 5–10% of all tibia fractures and are more often due to high-energy mechanisms of injury such as motor vehicle collisions, industrial accidents, and falls. Given the severity of injury, these have often been described as “explosion fractures” [2]. In addition to metaphyseal and intra-articular disruption, soft tissue injury is an important factor in the care of these injury patterns [3]. Due to the nature of these injuries, pilon fractures are associated with high complication rates, including postoperative stiffness, post-traumatic arthritis, delayed union, nonunion, infection, and persistent pain. A staged approach due to high consideration of the soft tissues is commonly undergone to avoid postoperative wound healing complications and reduce risk of infection [4].

Given the complexity of pilon fracture care, there is significant debate regarding fixation constructs to optimize patient outcomes [5,6,7]. Of particular interest is the timing and management of concomitant fibular fractures seen in these injuries. More recent studies have brought into question the necessity of fibular fixation in the treatment of pilon injuries [8]. Timing of fibular fixation has not been shown to play a significant role in the outcomes of pilon fractures, but did demonstrate an increase in the overall procedural time, regardless if performed at the index procedure or in a staged manner [9]. Current fixation options include open reduction internal fixation with plating, intramedullary screws, and external fixation.

Fixation of the fibula has been strongly advocated for in certain injury patterns. In particular, with pilon fractures having initial valgus angulation, restoration of fibular length and a lateral buttress can frequently aid in tibial plafond reduction and alignment. Similarly, fibular fractures involving the syndesmosis have often shown benefit from operative fixation [10,11]. Intramedullary fibular fixation has been described in the past decade in lateral malleolar fractures with success and has been shown to provide adequate fixation with successful union rates [12,13]. The attraction to this fibular intramedullary construct is its limited injury to soft tissues, when maintaining these structures is vital for healing in pilon injuries [14].

The current literature has evaluated several alternative fibular fixation methods in associated pilon fractures. However, there is little evidence comparing these methods in a single analysis and their overall influence on nonunion and malunion rates of either the fibula or tibia. The purpose of this study is to add to the paucity of the literature regarding fibular fixation methods in pilon fractures. In this study, we aimed to evaluate if fibular intramedullary fixation had superior outcomes regarding tibial union rates compared to open reduction internal fixation with plating. We hypothesized that a fibular intramedullary construct would result in noninferior tibial nonunion rate as compared to plating or nonoperative management of associated fibular fractures in complex pilon injuries.

## 2. Materials and Methods

This was a retrospective review comparing 107 consecutive patients with fractures of the tibial plafond with an associated fibula fracture admitted through our urban Level 1 trauma center from 1 January 2009 to 1 May 2019. Inclusion criteria were patients ≥ 18 years of age; a diagnosis of pilon fracture with an associated fibula fracture injury using ICD 10 codes S82.87, S82.40, S82.60; and underwent primary open reduction internal fixation procedures for pilon fractures based on procedure codes 0SHG04Z and 0SHF04Z or CPT codes 27827, 27826, 27814 by fellowship-trained orthopedic traumatologists, as well as at least 6 months clinical and radiographic follow up or to union if achieved prior to 6 months’ time. Exclusion criteria were patients with less than 6 months of follow ups; a history of prior ankle surgery; absence of fibula fracture; or who underwent definitive fixation with a primary ankle fusion, external fixator, tibiotalocalcaneal (TTC) nail, or primary limb amputation. This study was IRB approved and patient consent was obtained during timing of surgery as included in hospital standardized informed consent documentation.

Patients who met inclusion criteria for the study were allocated into one of the following treatment groups based on fibular fixation: fibular plating, fibular intramedullary device (including screw or nail), or no fibular fixation (control). The primary outcome of this study was tibial plafond union rates amongst varying types of fibular fixation. Additionally, the following information was gathered for each patient: age, sex, BMI, medical comorbidities, tobacco use, baseline ambulatory status, mechanism of injury, OTA/AO fracture classification, fibular fracture classification, presence and grade of open fractures according to Gustilo–Anderson, type of fixation for tibial and fibular fractures, necessity of syndesmotic fixation, postoperative complications, and necessity of an additional surgery. Additionally, clinical outcomes such as time to weightbearing and time to tibial and fibular union were obtained.

Radiographic analysis was performed by one of three of the authors on immediate postoperative plain films and compared by the same author to plain films at final follow up. Varus or valgus angulation was measured on full-length tibia or dedicated ankle X-rays with a line down the anatomic axis of the tibia and a line along the distal tibial articular surface, with malalignment or eventual malunion defined as a deviation ≥ 5° from the anatomic axis. The talocrural angle was measured in a standardized fashion utilizing the cobb angle from a line along the distal tibial articular surface and an additional line from the tips of the medial and lateral malleoli, as previously described [15]. In brief, a weightbearing mortise ankle X-ray with approximately 15° to 20° of internal rotation was obtained in clinic. A line was drawn from the tip of the medial malleolus to the lateral malleolus and a second line was drawn along the articular surface of the tibial plafond. The cobb angle between these two lines was the listed talocrural angle. These angles were measured by three authors in a standardized fashion to minimize measurement variability. Postoperative radiographs were analyzed for tibial or fibular nonunion defined as less than 3 bridging cortices visible on orthogonal radiographs at final follow up. Lastly, evidence of ankle degenerative joint disease was analyzed as determined by the Kellgren–Lawrence (KL) grading scale [16], which has been chosen by the World Health Organization as an acceptable reference standard. Grade 0 is defined as definite absence of radiographic changes of osteoarthritis. Grade 1 is defined as minute joint space narrowing with doubtful significance. Grade 2 is defined as definite osteophyte formation and possible joint space narrowing. Grade 3 is defined as moderate osteophyte formation with definite joint space narrowing, sclerosis, and possible deformity. Lastly, Grade 4 is defined as large osteophyte formation, significant joint space narrowing, sclerosis, and the possibility of joint deformity on both ends.

Statistical analysis was performed using Microsoft Excel (Version 2402), JASP (Version 0.17.1.0), and JAMOVI (Version 2.4.21.0). Descriptives were calculated for all variables. Means, standard deviations, and ranges were reported for continuous variables. Medians, confidence intervals, and ranges were reported for categorical variables. In order to compare groups, Student’s *t*-tests and ANOVAs were used for continuous variables, and chi-square tests were used for categorical variables. A significance level of *p* < 0.050 was set *a priori*, and *p*-values between 0.050 and 0.100 indicated trends.

## 3. Results

From our institution’s database, we identified 107 patients who met criteria for inclusion in this study over a 10-year span. We identified three groups of patients who underwent ORIF of their respective distal tibia fractures: Group A (pilon fracture fixation with fibular fracture fixation and plating, 19 patients), Group B (pilon fracture fixed with fibular fracture fixation treated with intramedullary implant, 23 patients), and Group C (pilon fracture fixation without fibular fixation, 65 patients).

The average age of our patient population in this study was 44 years old (range, 20 to 86 years old), consisting of 60 males (56.1%) and 47 females (43.9%). Baseline demographic characteristics are listed in Table 1. It should be noted there was a statistically significant prevalence of males in these cohorts, and the fibular plating group did have a decreased prevalence of smokers as compared to the other two cohorts. There were no further baseline demographic characteristic differences between the three groups with respect to age, medical history of diabetes, preambulatory status, or length of follow up.

Of the 107 patients included in this study, 42 patients (39.3%) underwent surgical stabilization of their fibula fracture. A summary of injury characteristics between the three groups is summarized in Table 2. There were no differences between the three groups with respect to tibial fracture OTA/AO grade or with respect to fibular fracture Weber grade. There was a total of 32 open tibial plafond fractures (29.9%). Patients were treated definitively for their distal tibia fracture 7.1 ± 11.7 days from injury, with 68 of 107 patients (63.0%) requiring temporization with an ankle spanning external fixator before definitive fixation.

The average length of follow up in this study was 66.0 weeks. At final follow up, collectively, 9 of 107 patients (8.4%) ultimately went on to develop a tibial nonunion. Additionally, 12 of 107 patients (11.2%) had a fibular nonunion amongst all three groups. There was a trend for decreased time to weightbearing in pilon fractures that did undergo fibular fixation (Group A and B) as compared to those that did not (Group C); however, this did not reach statistical significance (*p* = 0.066). There were no differences between groups with respect to time to tibial union (*p* = 0.982), incidence of tibial (*p* = 0.202) or fibular (*p* = 0.756) nonunion, as well as postoperative complications, as listed in Table 3.

We compared immediate postoperative alignment, alignment at final follow up, and subsequent radiographic evidence of ankle joint arthritis amongst the three cohorts (Table 3). Overall, there were no differences in postoperative alignment or instances of tibial malunion in any treatment group. There was no difference in postoperative or maintenance of fibular length with respect to if the fibula was fixed or the type of fibular fixation, as demonstrated by the talocrural angles. This was maintained from postoperative films to final postoperative follow up. There was no difference between the three groups with respect to presence of arthritis (KL grade ≥ 2 as initially defined). However, there was a statistical significance amongst the three groups with respect to KL grade amongst the treatment groups utilizing a chi-square analysis (*p* = 0.001). Furthermore, a subanalysis demonstrated a higher propensity for greater KL grade in fibula fractures that were not fixed (Group C) as compared to the groups that underwent fibular fixation utilizing plate fixation (Group A; chi-square analysis; *p* = 0.010) and screw fixation (Group B; chi-square analysis, *p* = 0.014), respectively.

## 4. Discussion

Treatment of complex pilon fractures remains a challenging problem for orthopedic surgeons, with many controversies regarding ideal management to optimize clinical outcomes in the trauma population. There is substantial consensus that ORIF and anatomic reduction in the tibial articular surface are paramount in preventing the onset of post-traumatic arthritis. Regardless, these injuries portend a guarded prognosis given the musculoskeletal insult and substantially negatively impacting functional outcomes and quality of life [17]. There is a significant variance in methodology to addressing the reduction in the articular surface of the distal tibia. Additionally, there is limited consensus on fibular management in pilon injuries with associated fibular fractures and how it influences outcomes clinically as well as radiographically.

Throughout the evolution of pilon fracture management, fibular fixation had been considered a staple in the staged management of these injuries [18,19]. Whether addressed at the time of application of external fixation or definitive management, fibular fixation was seen as a necessity by the treating surgeon [7,20]. Over time, subsequent investigations challenged this line of thinking, and Williams et al. [21] demonstrated increased complications with fibular fixation, negating addressing the fibula to maximize outcomes in pilon fractures.

Despite these interventions, pilon nonunion rates have been reported as high as 17% [22]. Recent literature has brought into question the necessity of fibular fixation, citing no difference in pilon union rates between operative or nonoperative management of fibular fractures. A study by Kurylo et al. [8] demonstrated no difference in radiographic alignment or achievement of union with complex high-energy tibial plafond fractures treated without fibular fixation. In this article, they emphasized the importance of articular reduction, joint line alignment, and appropriate fibular length to maximize clinical outcomes and radiographic union.

Our findings suggest that the presence or absence of fibular fixation did not increase the rate of nonunion in either the distal tibia or fibula in these pilon injuries (8.4%, *p* = 0.202 and 11.2%, *p* = 0.756, respectively). This study is one of the first to compare multiple methods of fibular fixation and how it pertains to radiographic healing. Fibular fixation may be incorporated by the treating surgeon to assist in maintaining reduction during the staged or final construct, without consequence as it relates to union rates in tibial plafond fractures. Several previous studies have cited the utility of fibular fixation in complex pilon injuries to prevent tibial nonunion and to provide a valgus buttress, stabilizing the lateral column [19,21]. In this study, the utilization of fibular fixation was left at the discretion of the treating surgeon, balancing soft tissue swelling, open wounds, and clinical acumen. It is the opinion of the authors that in the setting of initial valgus failure or significant medial column comminution, anatomic fibular fixation may serve as a helpful reduction aid for tibial plafond fractures and should be determined on a case-by-case basis.

Interestingly, there were no instances in postoperative malunion amongst any of the treatment groups in this study, regardless of if the fibula was fixed at final follow up. Additionally, there were no differences amongst talocrural angles amongst the three groups. In this study, the talocrural values amongst each group were on the lower end of normal, which may suggest inadequate restoration of fibular length, one of the four core principles initially described by Rüedi and Allgower in the management of pilon fractures. However, values were consistent across treatment arms, and with the standard deviations may reflect inter/intra-observer variability, and ultimately demonstrated no statistically significant difference, as these values were within normal tolerances. The importance of fibular alignment and length is important for functional outcomes following ankle injuries. The distal fibula remains critical in stabilization of the mortise and facilitating internal and external rotation during tibiotalar motion. With inadequate alignment and length, the resulting talar instability has been demonstrated to have altered ankle biomechanics, decreased functional outcomes, and accelerated ankle degeneration in ankle fractures [23]; however, this principle has never been directly studied in isolation with pilon fractures [24]. Talar shift as a result of fibular shortening has been shown to alter tibiotalar contact pressures by approximately 42% [25]. This relationship was further corroborated in a cadaveric study by Mason et al. [26] in which the distal fibula was progressively shortened in 5 mm increments. In their study, just under half of all ankles experienced lateral talar translation with 5 mm of shortening, and all specimens demonstrated significant lateral talar translation by 10 mm of shortening. An additional study demonstrated that fibular shortening greater than 2 mm, lateral translation, or external rotation greater than 5° was directly correlative to increased contact pressure onto the medial talar dome [27]. These data are further supported a prospective study that evaluated talocrural angle as a predictor for worse functional outcome in unstable ankle injuries [28]; however, there has not been any direct study evaluating postoperative talocrural angle and resulting PROs.

The Kellgren–Lawrence grading scale has been validated as an accurate and reproducible radiographic grading system for the assessment of ankle arthritis, with good inter- and intra-observer reliability and being predictive of clinical symptomatology [16] and has been well accepted as an adequate reference standard. In our study, the KL grading was used to compare fibular fixation methods in the absence of formally collected patient reported outcomes scores (PROs). Our cohort demonstrated a statistically significant difference amongst the three treatment groups, with a higher KL grade in pilon fractures without fibular fixation. Although tibial plafond and fibular union rates were unaffected based on type or absence of fibular fixation, this may suggest that the lack of stabilization of the lateral column may play a role in post-traumatic arthritis. Horisberger et al. [29] demonstrated latency time and post-traumatic osteoarthritis (OA) were impacted by fracture type and severity of ankle fractures, with intra-articular injuries revealing approximately half the latency time as extra-articular fractures. Patient factors such as older age also demonstrated shorter latency time to OA. Postoperative complications also influenced outcomes negatively, but fibular fixation was not a variable investigated in this analysis. Patient factors in this study were propensity matched, including age and fracture type amongst groups, so the influence of fibular fixation in pilon fractures may warrant future prospective investigation. It is important to note that in their study, they defined latency time as the time from initial injury to the development of clinical and radiographic end-stage ankle arthritis necessitating either ankle arthrodesis versus ankle arthroplasty. Pilons, in their study, on average had a reported latency time of 21.5 years (1–50 years). While their reported latency does extend past the average length of follow up of this study, their definition focuses solely on end-stage arthritis necessitating surgical intervention and does not fully capture the spectrum of development of post-traumatic arthritis and the clinical and functional pain and limitation that a patient may experience. This paradigm highlights the importance of accompanying PROs alongside these radiographic analyses, both presently and at extended time points postoperatively, to better characterize the impact these injuries have on patients’ function. Furthermore, the inclusion of PROs would help better quantify differences in clinical pain amongst varying KL grades of arthritis amongst the different treatment arms.

The highest priority must be maintained regarding soft tissues and skin bridge between incisions when planning definitive operative stabilization. Traditionally, the skin bridge width between incisions has been recommended to be at least 7 cm to reduce complications. However, multiple investigations have scrutinized these teachings. Howard et al. [30] demonstrated low complication rates with over 80% of their patients having incisions within 7 cm of each other. Additionally, Moore et al. [31] demonstrated no significant wound complications with skin bridges 2 cm and greater; however, these were in individuals undergoing elective foot and ankle surgery and may not directly correlate in the acutely traumatized soft tissue envelope. Full-thickness flaps and meticulous soft tissue handling is paramount to avoid skin edge injury and necrosis [24]. Skin bridge distances in multiply incised subjects in this investigation were beyond the scope of study. However, there were no differences in postoperative wound complications, superficial infection, or deep infection between groups in this study treated with plating versus an intramedullary implant.

In addition to wound complications, postoperative infection management in pilon fractures remains challenging. Complex pilon injuries frequently require extended time in the operating room to optimize fracture reduction and alignment. A study by Shafiq et al. [32] demonstrated that after surgical duration greater than 120 min, each additional 10 min of surgical duration for tibial fixation had an increased likelihood of necessitating postoperative incision and drainage, treatment of infection, and implant removal. There was a similar linear relationship for the management of concomitant fibular fractures in their study. Patients in this study who sustained superficial wound infections (15 of 107) were managed with antibiotics alone, and all patients went on to heal uneventfully. Deep infections reported in 7 of 107 cases necessitated removal of hardware, debridement, infection eradication, and definitive fixation of the tibia to promote union. Of the 7 patients with deep infection in this study amongst treatment groups, 6 patients were able to successfully achieve tibial union at final follow up. Similar to these data, a study by Faber et al. [13] evaluated the use of intramedullary fibular fixation in malleolar ankle fractures and its use in pilon fractures. In their study, there were no differences in complications between the two groups with similar union rates, wound complications, or necessity of implant removal.

There were several limitations to this paper, including its retrospective design. Postoperative radiographic analyses were performed, which may potentiate interobserver variability in interpretation. However, PROs were not formally collected and would certainly strengthen this data, which could further elucidate patient symptomatology in addition to radiographic changes. The KL grading scale was used as a radiographic parameter to compare groups in the absence of reliable PRO data. This was chosen to provide objective criteria that would be influenced by the fibular fixation method. Certainly, the addition of PROs would better elucidate specific clinical outcomes and impact in quality of life. As previously mentioned, with the concern for a longer latency period in the development of post-traumatic arthritis, longer term studies correlating KL grade, clinical symptoms, and PROs would provide useful prognostic information to educate and guide patient care. Further prospective studies with the inclusion of PROs are warranted to provide comprehensive analysis.

## 5. Conclusions

Treatment of complex pilon injuries remains a challenge for orthopedic surgeons with respect to construct and timing of operative fixation. These data suggest that treatment of fibular fractures with or without intramedullary fixation devices showed no difference in tibial or fibular nonunion rates in pilon fractures compared to open reduction internal fixation with plating. Therefore, discretion is left to the treating surgeon as to the necessity and method of fixation to assist in overall stability either during the staged or definitive fixation of pilon fractures.

## Figures and Tables

**Table 1:** Baseline patient demographics.

	Group A (19 Patients)	Group B (23 Patients)	Group C (65 Patients)	*p*
Age, Mean ± SD	42.7 ± 13.7	45.4 ± 13.2	44.6 ± 14.6	0.827
BMI, Mean ± SD	28.1 ± 6.7	28.8 ± 6.5	30.2 ± 10.0	0.582
Sex, n	M: 14 F: 5	M: 14 F: 9	M: 32 F: 33	0.050
Diabetes, n	0	1	8	0.061
Smoking, n	0	11	19	0.001
Osteoporosis, n	1	0	1	0.074
Peripheral Arterial Disease, n	1	0	0	0.196
Pre-injury Ambulatory Status				
No Assistive Devices, n	19	23	64	0.419
Cane, n	0	0	0	
Walker, n	0	0	1	

**Table 2:** Fracture characteristics of tibial plafond fractures for patients treated with ORIF versus no fixation of their fibula fracture.

	Group A (19 Patients)	Group B (23 Patients)	Group C (65 Patients)	*p*
Open Fractures, n (%)	8 (42%)	7 (30%)	17 (26%)	0.409
Grade 1	1	1	4	0.809
Grade 2	6	4	11	
Grade 3	1	2	2	
Fibula Fracture Classification				
Weber A	0	0	0	0.805
Weber B	7	9	26	
Weber C	12	14	39	
Tibial OTA/AO Classification				
43A	2	1	5	0.640
43B	7	14	32	
43C	10	8	28	

**Table 3:** Postoperative clinical and radiographic outcomes at final follow up.

	Group A (19 Patients)	Group B (23 Patients)	Group C (65 Patients)	*p*
**Clinical Outcomes**				
Time to Weightbearing, weeks	12.1 ± 6.3	12.4 ± 4.3	10.0 ± 4.5	0.066
Superficial Infection, n	3	4	6	0.510
Deep Infection, n	2	2	3	0.588
DVT, n	0	0	1	0.419
Time to Tibial Union, weeks	18.9 ± 5.4	18.6 ± 7.8	17.2 ± 8.4	0.982
Tibial Nonunion, n	3	3	3	0.202
Fibular Nonunion, n	3	2	7	0.756
Tibial Implant Removal, n	5	6	11	0.511
Fibular Implant Removal, n	3	2	0	0.046
**Radiographic Analysis**				
Talocrural Angle, postoperative	81.3° ± 4.5°	78.2° ± 4.7°	79.2° ± 4.8°	0.250
Talocrural Angle, final follow up	80.4° ± 4.4°	78.6° ± 3.8°	78.6° ± 9.9°	0.896
Ankle Degenerative Joint Disease Present, n	18	19	38	0.530
Kellgren–Lawrence Grading, n	Grade 0: 0	Grade 0: 0	Grade 0: 7	0.001
	Grade 1: 1	Grade 1: 4	Grade 1: 10	
	Grade 2: 5	Grade 2: 6	Grade 2: 19	
	Grade 3: 6	Grade 3: 5	Grade 3: 19	
	Grade 4: 7	Grade 4: 8	Grade 4: 10	

## Data Availability

The data presented in this study are available on request from the corresponding author due to hospital data stored in a secure folder within our institution for patient privacy.
